# Ileocolic venous thrombophlebitis from associated enteropathogenic *E. coli* infection

**DOI:** 10.1093/jscr/rjaf740

**Published:** 2025-09-14

**Authors:** Brendan P Stewart, Jen-Yuan C Kao, Kristina Kuklova, Marwan Alaoudi

**Affiliations:** Department of General Surgery, UConn Health Center, 263 Farmington Ave, Farmington, CT 06032, United States; UConn School of Medicine, 263 Farmington Ave, Farmington, CT 06032, United States; Department of General Surgery, UConn Health Center, 263 Farmington Ave, Farmington, CT 06032, United States; Department of General Surgery, The Hospital of Central Connecticut, Hartford Healthcare, 100 Grand St, New Britain, CT 06106 United States

**Keywords:** mesenteric thrombophlebitis, enteropathogenic *E. coli*, ileocolic thrombophlebitis

## Abstract

Enteropathogenic *Escherichia coli* (EPEC) is a pathogenic strain of *E. coli*. Mesenteric vein thrombophlebitis is of surgical concern, as there is a risk of the development of bowel ischemia. We present a unique case of a patient with EPEC diarrhea who subsequently developed ileocolic thrombophlebitis. Our patient presented to the emergency department with 6 days of diarrhea and abdominal pain. A computed tomography angiography (CTA) abdomen and pelvis was performed which revealed occlusion of the ileocolic vein with perivenous inflammation, consistent with mesenteric thrombophlebitis. A gastrointestinal polymerase chain reaction (GI PCR) was positive for EPEC. The patient was managed non-operatively with intravenous antibiotics and anticoagulation with resolution of symptoms. Ileocolic thrombophlebitis is a rare cause of acute abdominal pain. Our patient is one of the first documented cases to develop thrombophlebitis following EPEC infection. We hypothesize the EPEC infection and inflammation predisposed our patient to developing ileocolic thrombophlebitis.

## Introduction

Enteropathogenic *Escherichia coli* (EPEC) is a pathogenic strain of the *E. coli* bacteria with virulence factors capable of causing acute diarrheal infections [1]. While EPEC is known to cause acute pediatric diarrheoa, it is much less common for adults to present with EPEC-associated diarrheal illness [2, 3]. Several case reports have documented various infectious etiologies associated with mesenteric thrombophlebitis, with the two most common causes being acute diverticulitis and appendicitis [4–8]. Both conditions are associated with an enteric gram-negative bacterial infection, similar to EPEC.

Mesenteric vein thrombophlebitis is of particular surgical concern, as there is a risk of the development of bowel ischemia and necrosis that requires acute intervention [9, 10]. The ileocolic branch of the superior mesenteric vein drains the distal ileum, cecum, and ascending colon. Ileocolic thrombophlebitis is a rare phenomenon that has been documented in case reports in the context of COVID-19 infection, appendicitis, and right-sided diverticulitis [11–13]. In these patients, diagnosis of thrombophlebitis was established through computed tomography (CT) imaging showing filling defects in the ileocolic vein. We present a unique case of a patient with EPEC diarrhea who subsequently developed ileocolic thrombophlebitis.

## Hospital course

Our patient is a 79-year-old male with past medical history remarkable for hypertension and prior superior mesenteric venous (SMV) thrombosis 7 years prior to presentation, not currently on anticoagulation, who presented to the emergency department with 6 days of diarrheoa and abdominal pain. Patient reports he had onset of gastrointestinal symptoms with nausea, vomiting, and diarrhea over the prior 6 days, with increasing fevers, chills, and abdominal pain leading to presentation to the emergency department. He had no prior surgical history and had no tobacco, alcohol, or illicit drug use. He reports he was on oral anticoagulation for 6 months following diagnosis of his prior SMV thrombosis, but did not follow up after completion of treatment and did not have any additional abdominal pain related to his thrombosis.

**Figure 1:**
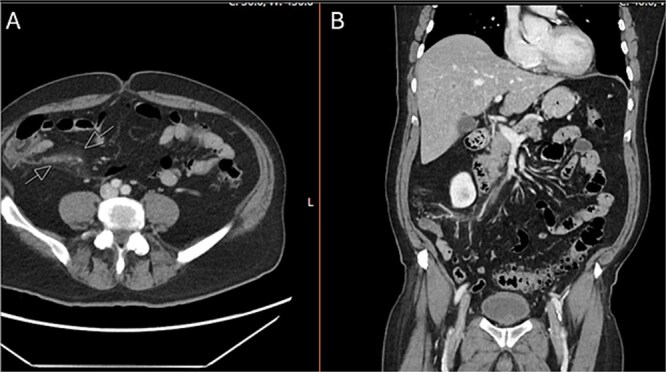
CT abdomen and pelvis with IV contrast. A) Axial and B) coronal series identifying normal appendix and right lower quadrant mesenteric inflammatory changes.

Upon arrival to the emergency department, the patient was hemodynamically stable and afebrile. On exam, the patient’s abdomen was soft, mildly distended, and tender in the right lower quadrant. He was noted to have a white blood cell count to 22.1, hemoglobin/hematocrit of 14.3/46.0, and platelets of 187. He had a lactic acidosis to 2.8. His electrolytes were unremarkable. CT scan of the abdomen and pelvis revealed no free air or free fluid, small amount of cecal wall thickening, and mild infiltrative changes within the mesenteric fat in the right lower quadrant with associated small mesenteric nodes with a normal-appearing appendix (Fig. 1).

**Figure 2:**
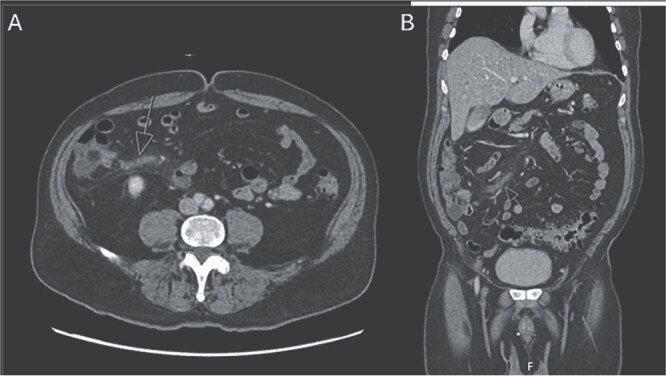
CTA abdomen and pelvis venous phase. A) Axial and B) coronal series revealing filling defect within the ileocolic vein with surrounding inflammatory changes, consistent with mesenteric thrombophlebitis of the ileocolic vein.

The patient was admitted to the surgery service for resuscitation and close monitoring. Given his prior thrombotic event, the patient was started on a continuous heparin infusion, anti-Xa goal 0.3–0.7. The patient received 2 L of intravenous fluids as well as a continuous fluid infusion of lactated ringers at 100 cc/hr. The surgery team performed serial abdominal exams which remained stable. Additionally, he was started on prophylactic ceftriaxone and metronidazole. A CTA abdomen and pelvis was subsequently performed, which revealed occlusion of the ileocolic vein with perivenous inflammatory change, consistent with mesenteric thrombophlebitis (Fig. 2). The celiac trunk, superior mesenteric artery, renal arteries and inferior mesenteric artery were all patent. Venous phase imaging showed patent portal vein and patent main superior mesenteric vein. On hospital day (HD) 1, the patient’s lactic acidosis cleared. A GI PCR was obtained which was positive for enteropathogenic *E. coli* (EPEC). Hematology and Oncology was consulted and recommended continued therapeutic anticoagulation and outpatient oral anticoagulation. The patient had a short period of respiratory distress on HD 2, attributed to be secondary to volume overload, and responded appropriately to diuretics. On HD 3, the patient’s leukocytosis resolved, and he was slowly advanced to a regular diet. On HD 4, the patient was discharged on remaining course of amoxicillin-clavulanic acid (Augmentin). After discharge, the patient’s blood cultures returned positive for *Fusobacterium nucleatum* and his Augmentin course was extended for an additional seven days.

## Discussion

Ileocolic thrombophlebitis is a rare cause of acute abdominal pain. Our patient is one of the first documented cases to develop thrombophlebitis following EPEC diarrhea infection.

Prior case reports of ileocolic thrombophlebitis have described pain in the right lower quadrant (RLQ) similar to acute appendicitis [11, 14]. Chou *et al.* describes how their patient was recovering from COVID-19 infection when he developed RLQ pain, thus obtaining a CT which showed mesenteric thrombophlebitis and lymphadenitis. Similar to our patient, he was treated with anticoagulation and antibiotics. Sirecki *et al*. also describes a patient who presented with fever and abdominal pain who was subsequently found to have ileocolic venous thrombophlebitis on CT imaging. Again, like our patient, this patient was treated non-operatively with anticoagulation and antibiotics [14].

Risk factors for mesenteric thrombosis include injury, stasis, malignancy, infections, trauma, inherited coagulopathies, and inflammation [15]^.^ We hypothesize that our patient developed inflammation from the EPEC diarrheoa, which combined with the infection, pre-disposed our patient to developing thrombophlebitis. He did not exhibit any coagulopathies, no recent trauma, no malignancy, and no recent immobilization or stasis. Additionally, our patient did show bacteremia, which could have been from his initial EPEC diarrheoa illness or his thrombophlebitis. Unfortunately, blood cultures resulted after the patient was discharged, so our patient’s antibiotic course was extended, with resolution.

In conclusion, we report a case of ileocolic thrombophlebitis associated with EPEC diarrhea. Our patient was managed non-operatively with anticoagulation and antibiotics. Our patient highlights the importance of evaluating all possible etiologies for abdominal pain, as ileocolic venous thrombosis is much rarer compared to other infectious etiologies. In obtaining comprehensive history and physical exam, risk factors for thrombophlebitis can be determined, and early initiation of anticoagulation can help prevent sequela of mesenteric venous thrombosis.
